# Neonatal microbial colonization in mice promotes prolonged dominance of CD11b^+^Gr-1^+^ cells and accelerated establishment of the CD4^+^ T cell population in the spleen

**DOI:** 10.1002/iid3.70

**Published:** 2015-06-18

**Authors:** Matilde B Kristensen, Stine B Metzdorff, Anders Bergström, Dina S M Damlund, Lisbeth N Fink, Tine R Licht, Hanne Frøkiær

**Affiliations:** 1 Department of Veterinary Disease Biology, Faculty of Health Medical Sciences, Section of Experimental Animal Models, University of Copenhagen1870 Frederiksberg C, Denmark; 2 Department of Food Microbiology, National Food Institute, Technical University of Denmark2860 Søborg, Denmark; 3 Novo Nordisk, Novo Allé2880 Bagsværd, Denmark

**Keywords:** CD11b^+^Gr-1^+^ myeloid cells, CD4^+^ T cells, microbiota, neonatal hematopoiesis

## Abstract

To assess the microbial influence on postnatal hematopoiesis, we examined the role of early life microbial colonization on the composition of leukocyte subsets in the neonatal spleen. A high number of CD11b^+^Gr-1^+^ splenocytes present perinatally was sustained for a longer period in conventionally colonized (CONV) mice than in mono-colonized (MC) and germfree (GF) mice, and the CD4^+^ T cell population established faster in CONV mice. At the day of birth, compared to GF mice, the expression of *Cxcl2* was up-regulated and *Arg1* down-regulated in livers of CONV mice. This coincided with lower abundance of polylobed cells in the liver of CONV mice. An earlier peak in the expression of the genes *Tjp1*, *Cdh1*, and *JamA* in intestinal epithelial cells of CONV mice indicated an accelerated closure of the epithelial barrier. In conclusion, we have identified an important microbiota-dependent neonatal hematopoietic event, which we suggest impacts the subsequent development of the T cell population in the murine spleen.

## Introduction

During the past decades, the prevalence of allergies and autoimmune diseases has increased dramatically in the western world 1, and it is generally accepted that the microbial environment is a key player in the development of these disorders 2,3. It is, however, still not clear how and when the intestinal microbiota influences the development of an immune system that is more prone to developing immune-related diseases as opposed to development of a healthy immune system.

In 2008, a meta-study concluded that delivery by caesarian section significantly increases the risk of developing childhood-onset type 1 diabetes 4, and a large long-term birth-cohort study of early life intestinal microbiota associates reduced fecal microbial diversity in early life with increased risk of later development of allergic diseases 5. Recently, also maternal antibiotics use during pregnancy has been linked to an increased risk of early childhood asthma 6. Altogether, these studies indicate that the microbiota in early life influences disease development. This is supported by several studies describing mode of delivery and neonatal feeding as highly influential on gut microbiota composition in early life as well as on permanent changes of the microbial community in the gastrointestinal (GI) tract 7,8. Hence, events during perinatal life, affecting the first microbes that inhabit the epithelium at mucosal sites, may be determining for the “set-point” of the immune system.

Several studies have suggested a higher intestinal permeability in neonates than in adults. Intestinal integrity establishes rapidly and concomitantly to introduction of oral feeding in early life but can be influenced by infant feeding (breastfeeding vs. formula) and time of birth (preterm vs. term) 9,10. Recently, microbial products were shown to translocate from mucosal surfaces to central lymphoid organs and specifically enhance systemic innate immune responses in mice 11,12. Though it remains speculative, the neonate intestine, due to its lowered integrity, could be particularly susceptible to translocation of commensal bacteria or microbial products across the intestinal barrier, which successively may influence the induction of mucosal and systemic tolerance in neonates.

We have recently reported that colonization with a complex microbiota, but not with a single bacterial strain, affected the adaptive immune development in neonatal mice, and that this response was accompanied by reduced proinflammatory cytokine gene expression in the intestine of conventionally colonized (CONV) animals 13. A diverse microbiota has been shown to be indispensable for the adaptive immune system, as colonization with a conventional microbiota but not mono-colonization with *Escherichia coli* or lactobacilli supports development of oral tolerance in mice 14. Additionally, germfree (GF) mice have been shown to have fewer Tregs in lymphoid organs than CONV mice, and the regulating effect of the GF-derived Tregs was furthermore shown in vitro to be impaired compared to CONV Tregs 15. Recently it was shown that postnatal microbial colonization plays a prominent role in the induction and establishment of a group of neutrophil-like cells with B cell-helper function in the marginal zone of the neonatal spleen, hereby promoting an antimicrobial immunoglobulin defense by interacting with B cells 11. Thus, by influencing Tregs in lymphoid organs and production of immunoglobulins in spleens of newborns, it seems that the commensal microbiota holds a key role in priming and shaping of the early adaptive immune system. The mechanism behind this influence of postnatal colonization on the development of systemic immunity is, however, largely unknown.

Integrin CD11b is expressed on several leukocytes, including mature and differentiating monocytes and granulocytes, and CD11b^+^ cells in neonatal mice is therefore expected to constitute a heterogeneous group of cells. CD11b is involved in inhibition of TLR signaling 16 and subsets of neutrophils, characterized by high expression of CD11b and Gr-1, have been shown to hold regulatory properties and be involved in down-modulation of T cell responses 11,17 Thus, these cells might constitute a key regulator in establishment of the adaptive immune system.

The bone marrow (BM), spleen, and liver cooperatively contribute to the hematopoietic homeostasis in the neonatal period. Perinatally, hematologic stem cells (HSCs) present in the liver migrate to the BM where they remain throughout life but a proportion of the hematopoietic liver cells migrate to the spleen or, upon circulation, homes to the liver 18,19. LPS stimulation of splenocytes results in a fast and transient up-regulation of the two chemokines; *Cxcl1* (KC) and *Cxcl2* (MIP-2) expression 20 and importantly, a transient induction of MIP-2 in vivo leads to not only fast recruitment of polymorphonuclear leukocytes into the peripheral blood but also rapid activation of HSCs and up-regulation of CD11b on these cells 21.

The present study is based on the hypothesis that early microbial colonization of the GI tract leads to influx of microorganisms from the gut into circulation. We hypothesized that this affects the subsequent composition of spleen cells, and that this is of importance for establishing a well-balanced immune system. We examined the development of cell subsets in the spleens of pups born by GF dams, *Lactobacillus acidophilus* NCFM mono-colonized (MC) dams, and CONV dams, and found that the establishment of the CD4^+^ T cell pool in the spleen was accelerated by conventional microbiota. The largest difference between CONV and GF mice was seen in the very first days after birth, where the proportion of neutrophil-like cells positive for the markers CD11b^+^ and Gr-1^+^, naturally present as part of the perinatal pool of myeloid progenitor cells in spleen and liver, remained high during the first week in spleens of mice born by CONV dams while dropping rapidly after birth in spleens from GF mice. We suggest that the longer-lasting high number of CD11b^+^Gr-1^+^ cells is an important microbiota-dependent postnatal hematopoietic event that influences the subsequent development of adaptive immunity.

## Results

### The microbiota affects the cell composition in spleen

**Figure 1:**
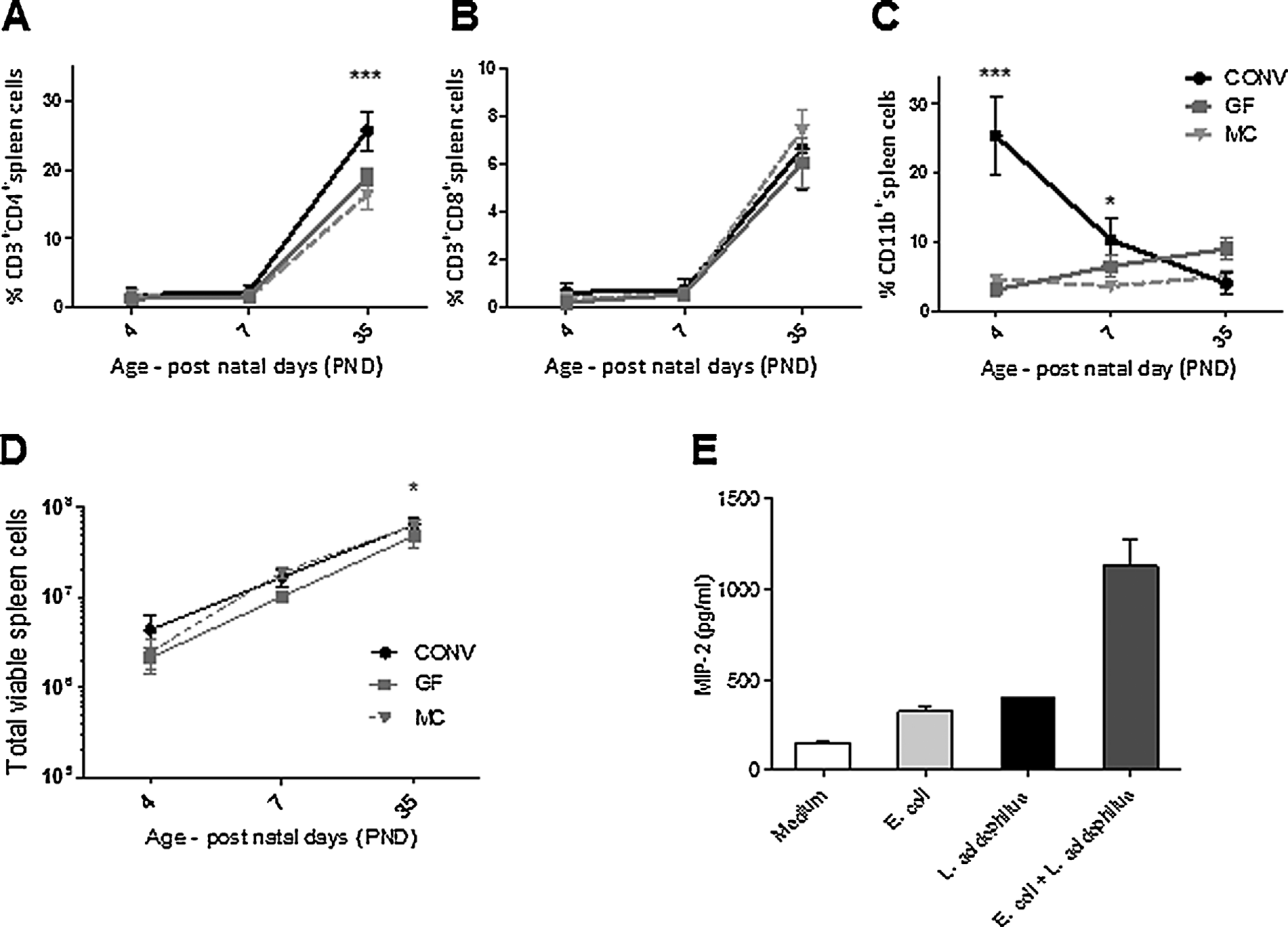
The establishment of the CD3^+^CD4^+^ population is accelerated by the microbiota and is preceded by dominance of CD11b^+^ cells in the spleen of newborn CONV mice. (A) Time-dependent development of spleen cell populations at PND4, PND7, and PND35 in GF, MC, and CONV mice, analyzed by flow cytometry for markers (A) CD3 andCD4, (B) CD3 and CD8, and (C) CD11b. Data are expressed as percentages of total viable cells and analyzed by two-way Anova with Bonferroni post test, ***p < 0.001, *p < 0.05, mean ± SD, each point on graphs represents 3–7 individual animals per group per timepoint. (D) Time course of the absolute number of viable cells in spleens of CONV, GF, and MC mice, *n* = 2–5 animals for each data point. Data are analyzed by two-way Anova with Bonferroni post test, *p < 0.05, mean ± SD. (E) Release of MIP-2 from splenocytes isolated from CONV mice on PND4 and cultured with *L. acidophilus* NCFM, *E. coli* Nissle 1917, or a mixture of both bacteria, *n* = 2 animals, mean ± SD.

To assess the influence of the microbiota on the cellular composition in the developing spleen, we measured the proportion of various leukocyte subsets in spleens of mice aged 4, 7, and 35 days born by CONV dams, GF dams, and MC dams, respectively (Fig. 1A–C). In the period of 7–35 days after birth, the proportion of CD3^+^CD4^+^ T cells in spleens of all colonization groups increased from approximately 2% at postnatal day 7 (PND7) to 16–25% of the total splenocytes on PND35 (Fig. 1A). While no differences were seen at PND35 for the number of CD3^+^CD8^+^ cells in the three groups (Fig. 1B), the number of CD3^+^CD4^+^ cells was significantly higher in the CONV group (25.7%) as compared to the MC group (16.3%) and the GF group (19.0%) (Fig. 1A). At later time points, this difference diminished, and no differences were seen between the CONV and GF T cell levels in adult mice (results not shown). There were no significant differences at any timepoint between the three colonization groups for CD19^+^, CD49b^+^, or CD11c^+^ spleen cells (results not shown).

Remarkably, a large proportion of CD11b^+^ cells (25.4% of all viable splenocytes) characterized spleens from PND4 CONV mice, while the CD11b^+^ cells only constituted mean levels of 3.1 and 4.6% in GF and MC PND4 mice, respectively (Fig. 1C). The high proportion of CD11b^+^ cells at PND4 diminished to 10.3% of total spleen cells at Day 7 in CONV pups, but CONV spleens still held significantly higher levels of CD11b^+^ cells than attained in MC pups. The total numbers of viable cells were significantly lower in GF compared to the CONV and MC groups at PND35. No differences were seen between CONV and MC at PND35 (Fig. 1D).

The striking lack of effect of mono-colonization on the cellular development of the neonatal spleen prompted us to investigate, whether there is a need for polymicrobial stimulation to regulate the expression of chemokines involved in recruitment of CD11b^+^ cells to the perinatal spleen. Consequently, we isolated spleen cells from CONV PND4 mice and cultured with *L. acidophilus*, *E. coli*, or a mixture containing half the concentration of each bacterium, to identify their ability to produce MIP-2 upon this stimulation. The splenocytes released markedly more MIP-2, when stimulated with *E. coli* and *L. acidophilus* in combination compared to stimulation with either of the bacteria in a monoculture (Fig. 1E), supporting a requirement for polymicrobial stimulation for recruitment of CD11b^+^ cells to the spleen.

Thus, the microbiota influenced the composition of the spleen both in the very early postnatal period, prolonging the presence of a large group of CD11b^+^ cells and significantly increasing the levels of CD4^+^ T cells during the later postnatal development of the CONV spleen. The nature and complexity of the microbiota furthermore strongly influenced the ability of spleen cells to produce MIP-2 important for recruitment and differentiation of CD11b^+^ cells.

### The high proportion of CD11b^+^Gr-1^+^ cells in the neonatal murine spleen is maintained by the presence of microbiota

**Figure 2:**
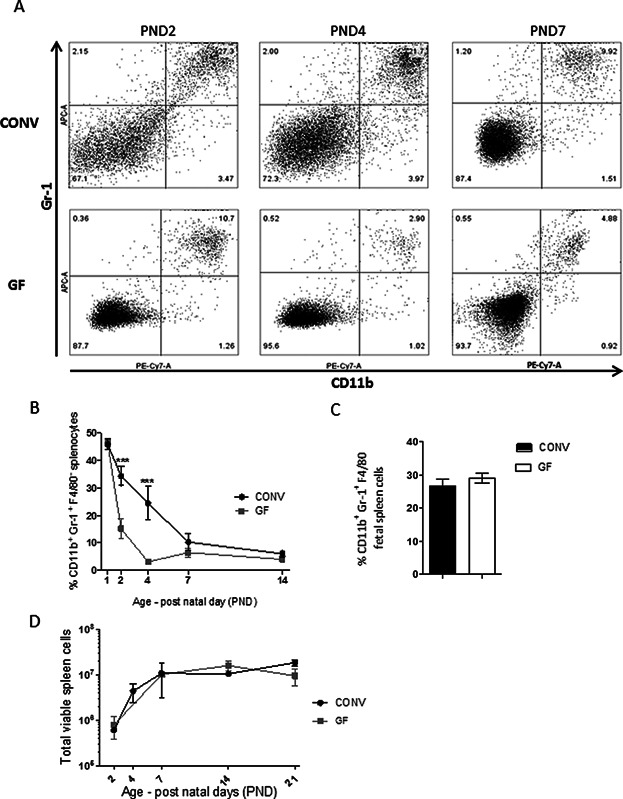
Microbial colonization prolongs the predominance of CD11b^+^Gr-1^+^ cells in the neonate spleen of mice during the first week of life. (A) CONV and GF splenocytes stained for surface markers CD11b and Gr-1 on PND2, 4, and 7. Plots represent cellular percentages of all viable cells (B) Time course for CD11b^+^Gr-1^+^ splenocytes in CONV and GF mice PND1 to PND14, data are analyzed with two-way Anova and Bonferroni post test, *n* = 3–5 animals, ***p < 0.001, mean ± SD. (C) Level of CD11b^+^Gr-1^+^ cells in spleens of fetal CONV and GF mice (PND-1). Columns present mean of two individual experiments in each group, each contains pooled spleens from 12 pups, mean ± SD. (D) Time course of the absolute number of viable cells in spleens of CONV and GF mice aged PND1 to PND21, *n* = 2–5 animals for each data point, mean ± SD.

Splenocytes from newborn mice were stained with anti-CD11b and anti-Gr-1 to investigate subsets of neutrophils, and F4/80 was included as macrophage marker. The CD11b^+^Gr-1^+^ group represented by far the majority of the CD11b^+^ cells described in fetal and neonate spleens (Fig. 2A) and was negative for the macrophage marker F4/80 throughout the experiment (data not shown). The CD11b^+^Gr-1^+^ cells were present in CONV as well as in GF spleens at Day 19 of gestation (PND-1) in comparable amounts (25–30%) and at the day of birth (PND1) the amount raised to as much as 46–47% of total viable spleen cells in both groups (Fig. 2B). As expected from the previous result for marker CD11b (Fig. 1A), the presence of microbial colonization strongly influenced the ability to maintain the large group of CD11b^+^Gr-1^+^ cells in the neonatal spleen after the day of birth. As early as PND2, the level of CD11b^+^Gr-1^+^ cells in GF mice decreased to 15% while the level in the CONV spleens only decreased to 34% on PND2. This difference between the groups was maintained in 4-day-old mice, as 24.5% of the cells of CONV spleens were CD11b^+^Gr-1^+^ cells compared to 3.1% of the cells in GF spleens (Fig. 2A, B). Importantly, we did not at any time point observe differences in the total number of cells in the spleens, which increased from below 10^6^ cells at PND2 to around 10^7^ at PND21 in both GF and colonized pups (Fig. 2C).

**Figure 3:**
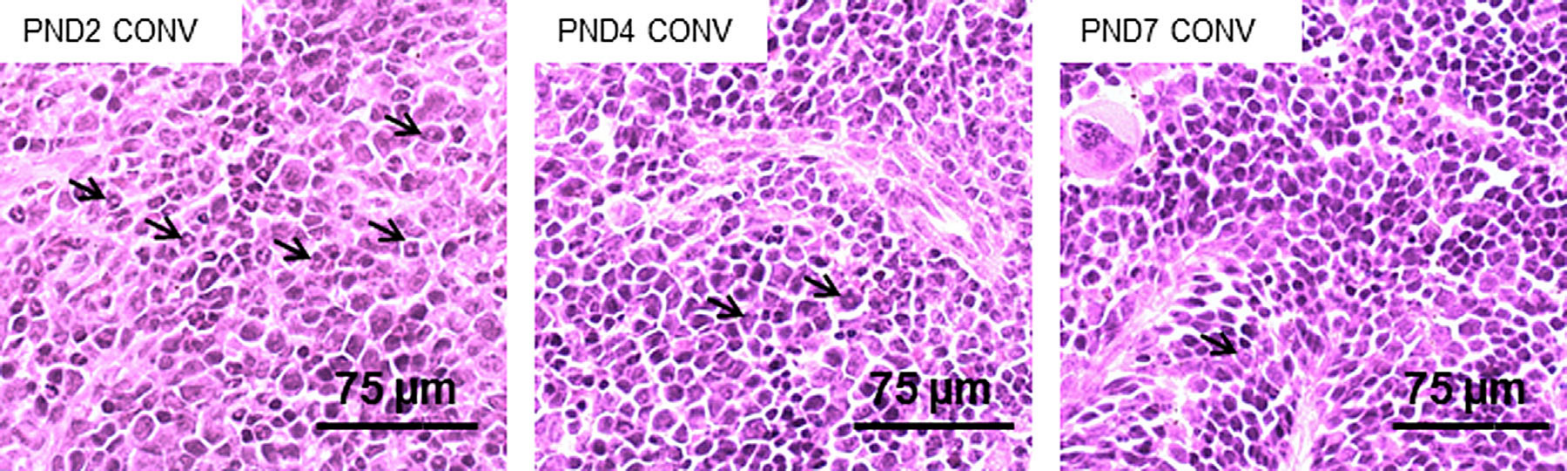
The number of granulocytes in the neonatal spleen is gradually reduced during the first 7 days of life. H&E stained tissue sections of CONV spleens. Arrows indicate polylobed or ring-shaped nuclei representative of mature or maturing granulocytes in the spleen tissue. Magnification, ×40. Data are representative for two independent experiments with 2–3 samples per group.

Of note, splenocytes of neonate CONV and GF animals comprised at least three distinctly labeled subpopulations with regard to forward and side scatter (FSC and SSC) and surface markers CD11b and Gr-1 (Supporting Information). Already at PND-1, the CD11b^+^Gr-1^+^ splenocytic subgroups differed between GF and CONV mice, as the single dominating subpopulation in GF mice changed from FSC^high^ to FSC^low^ from PND-1 to PND1, while cells of CONV mice sustained both subgroups of CD11b^+^Gr-1^+^ splenocytes, suggesting an influence of microbial colonization on immune development already during gestation. H&E staining of splenic tissues from PND2, 4, and 7 CONV mice (Fig. 3) showed decreasing numbers of cells with polylobed or ringed nuclei in CONV mice from Day 2 (71.0 ± 25.5) to Day 4 (19.9 ± 3.5) and Day 7 (4.1 ± 1.5) after birth; a morphology similar to mature and maturing neutrophils 20,22. This demonstrate that over the course of the first 7 days after birth, the cells with polylobed and ringed nuclei were less abundant in the spleen tissues at PND 7 compared to Days 2 (*p* = 0.0004) and 4 (*p* < 0.0001).

Taken together, the CD11b^+^ cells exhibited a phenotype of granulocytes, either conventional neutrophils or immature granulocytes, and constituted a significantly higher proportion of the spleen of CONV neonatal 2 to 4-day-old mice compared to older mice and to GF mice of the same age.

### Expression of Cxcl2 is up-regulated in the liver of colonized neonatal mice

**Figure 4:**
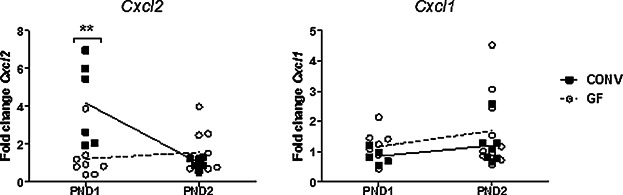
Microbial colonization is associated with a higher *Cxcl*2 expression in the liver at PND1. Expression of *Cxcl1 and* *Cxcl2* in livers of CONV and GF PND1 and PND2 mice determined by qPCR, *n* = 5–10 mice per group/day, significant difference is between the expression in GF and CONV livers of the same age, data are analyzed with one-way Anova and Bonferroni post test, **p < 0.01.

To address whether chemokine expression in the liver is altered in GF compared to CONV mice immediately after birth, we measured the expression of *Cxcl1* and *Cxcl2* in GF and CONV livers at PND1-2. As depicted in Figure 4, the expression of *Cxcl2* was significantly higher for pups of CONV dams than of GF dams on PND1. At PND2, no difference was seen. Expression of the *Cxcl1* in livers of GF and CONV neonate mice did not reveal any difference. GM-CSF and G-CSF likewise stimulate migration of neutrophils as well as the up-regulation of CD11b 21. There was no measurable gene expression for these cytokines in the liver (data not shown).

**Figure 5:**
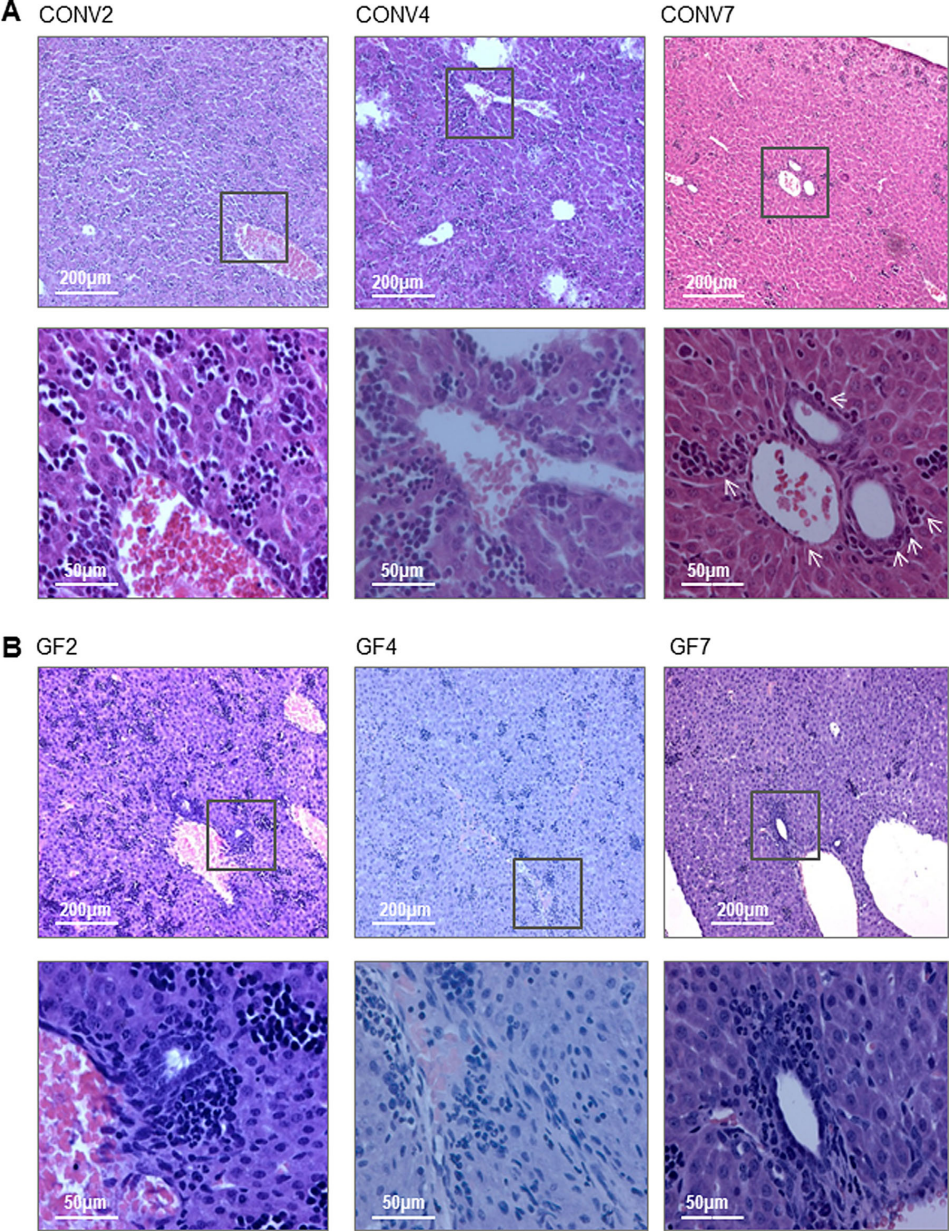
Microbial colonization leads to reduced level of neutrophil-like cells in livers of 2 to 4-day-old mice. H&E staining of neonatal liver tissue sections from CONV (A) and GF (B) mice at PND2, 4, and 7. Neutrophil-like cells accumulate around blood vessels at PND2 and 4 in liver tissues of GF mice, while these cells are largely absent from CONV liver tissues at the same age. Inserts depict cellular content around portal vein areas of liver tissues. From two independent experiments with 2–3 samples per group, four to five livers of each day and colonization were dissected and stained. The presented images are representative of all stains of the particular day and colonization, 10× and 40× magnification.

H&E stains of neonatal CONV and GF liver tissues (Fig. 5A, B) revealed that livers of CONV PND2 contained “clusters” of hematopoietic tissue at PND2, which decreased over PND4 and 7 (Fig. 5A, CONV2), and only low numbers of granulocytes of mature or immature granulocytic morphology were present in CONV liver tissues at PND2 (6.9 ± 3.5), 4 (17.0 ± 10.6), and 7 (24.9 ± 10.4) (Fig. 5A). A number of granulocytes with polylobed and ring-shaped nuclei tended to accumulate in vascular areas at PND7 in CONV liver tissues (Fig. 5A, right). In the GF livers, the hematopoietic tissue was markedly more distinct and abundant on PND2 compared to livers of CONV mice. Along with this, the GF liver tissue was richer in granulocytes with polylobed and ring-shaped nuclei at PND2 (52.0 ± 20.8) and PND4 (42.8 ± 17.7) compared to the CONV liver tissue of same age: PND2 (*p* = 0.0014) and PND4 (*p* = 0.0232). Most importantly, granulocytic cells were abundant around the portal veins in the GF livers on PND2 and PND4 (Fig. 5B). At PND7, the granulocytic cells were less abundant in the tissues and now lining the vessels of GF livers, but with similar numbers (47 ± 15.3) as for GF PND2 and PND4.

**Figure 6:**
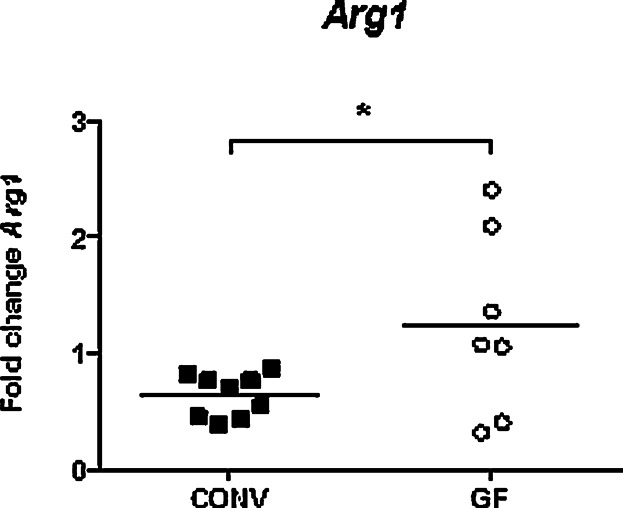
Expression of *Arg1*, a marker for immature granulocytes, is lower in PND1 livers of mice colonized with a conventional microbiota. Expression determined by qPCR in livers of CONV and GF mice on PND1, n = 7–9 mice per group, data are analyzed with Student's *t*-test (unpaired, two-tailed), *p < 0.05.

Cells of the CD11b^+^Gr-1^+^ phenotype as well as HSCs are known to express arginase-1 (*Arg1*) 11,23, and we consequently measured the expression of *Arg1* in livers of PND1 CONV and GF mice to support the observed differences in granulocyte and hematopoietic cellular content of the neonate livers. Interestingly, expression of *Arg1* was down-regulated in CONV mice as compared to GF mice (Fig. 6) indicative of a rapid drop in granulocytic cells and HSCs in the CONV neonate liver on PND1.

In summary, a significantly higher transcriptional level of *Cxcl2* in PND1 livers of CONV compared to GF mice indicated systemic influence of microbiota on the mobilization and activation of polymorphonuclear leukocytes at the day of birth. Histology of CONV and GF livers revealed distinctly more hematopoietic tissue in GF livers than in CONV livers on PND2, concomitantly with an accumulation of cells with polylobed and ring-shaped nuclei in the tissue and surrounding the blood vessels of PND2 and PND4 GF liver tissues. Opposite this, very few cells with similar morphology were observed in CONV liver tissues at PND2 and PND4.

### The microbiota influences the regulation of intestinal tight junction genes in the neonatal mouse

To investigate the possible role of the microbiota on expression of genes involved in intestinal permeability, we measured the expression of the tight junction genes; Tight junction protein 1 (*Tjp1 or Zo-1*), E-cadherin (*Cdh1*), and Junctional adhesion molecule A (*JamA*) in the distal ileal tissues of differently colonized mouse pups. The expression levels and the proteins encoded by these genes have previously been demonstrated to reflect the integrity of the intestinal barrier 24–26.

**Figure 7:**
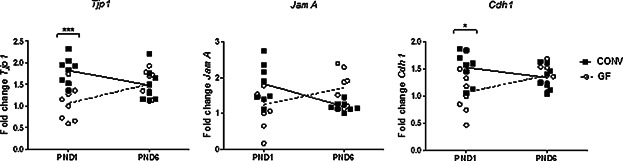
Microbial colonization increases expression of the tight junction (TJ) genes *Tjp1*, *Cdh1*, and *JamA* in ileum on the day of birth (PND1). Tight junction gene expression determined by qPCR in CONV, MC, and GF mouse pups on PND1 and PND6. *n* = 8 animals per group, data are analyzed with one-way Anova and Bonferroni post test, *p < 0.05, ***p < 0.001.

As shown for the expression of *Tjp1*, *JamA*, and *Cdh1* (Fig. 7), a higher relative expression of the genes in the ileum of CONV animals at PND1 as compared to the ileum of GF mice could be demonstrated. Moreover, the expression of all genes decreased from PND1 to PND6 in CONV intestinal tissue, while the expression increased from PND1 to 6 in the GF mice pups. In *L. acidophilus* MC ileal tissues, all three genes were on PND1 measured to a transcriptional level in between the higher expression levels in CONV neonate intestine and lower levels of the GF intestinal tissue and did not change over time (data not shown).

Taken together, these data suggest a difference in the regulation and maturation between the colonized and non-colonized intestine, and hence that the postnatal microbiota influences the kinetics of the maturation of the intestinal barrier.

## Discussion

A key role of the microbiota for proper development of a well-balanced immune system is generally accepted, but an understanding of how this development is supported by the microbiota is lacking. Here, we focused on hematopoietic events in the perinatal period and showed that very early postnatal events in the hematopoietic system comprising the liver, spleen, and BM are greatly influenced by the presence of microorganisms, and that microbial colonization at birth accelerates the establishment of the CD4^+^ T cell pool in the spleen during the first weeks of life in mice.

We demonstrated that the spleen of newborn mice, independently of the presence of microorganisms, contained high numbers of CD11b^+^Gr-1^+^ cells, which already at the day after birth (PND2), dropped dramatically. The CD11b^+^Gr-1^+^ cells decreased to the level of adult mice at PND4 in GF mice, while this number in pups of CONV mice decreased at a much slower rate and did not reach the adult level before 1–2 weeks of age. Whether the presence of these cells in the spleen is due to differentiation of HSCs present in the spleen at birth or reflects an influx of cells from other organs cannot be finally concluded from the present study. However, a previous study of the perinatal hematopoiesis in mice concluded that a significant proportion of HSCs and differentiating HSCs from liver migrate to the spleen 18. That the cells derive from the liver, rather than from proliferating HSCs in the spleen, is supported by our microscopy data of the CONV spleen showing abundant neutrophil-like cells with a polylobed or ring-shaped nucleus but only sparse presence of hematopoietic tissue. Moreover, in the livers from GF and CONV mice the hematopoietic tissue was easily distinguished, but remarkably more abundant and distinct in the neonate GF livers. The neutrophil-like cells were frequently distributed in the tissue and in particular accumulated around the portal vein areas in the GF livers, while in livers from CONV mice the differentiating neutrophil-like cells were notably fewer around the portal veins and almost absent in the tissue. The absence of neutrophil-like cells may indicate a greater early life efflux of the immature neutrophil-like cells from the liver of newborn pups of CONV mice coinciding with an increased influx of these cells to the spleen at PND2–4. The significant reduction in the expression of *Arg1* in the liver of CONV mice at the day of birth further supports an efflux of neutrophil-like cells as the expression of *Arg1* has been ascribed as unique for differentiating, yet immature myeloid cells as well as neutrophils in early life 11,17, and thus may reflect the abundance of neutrophil-like cells at this time point. Interestingly, in the spleen from fetuses taken from both CONV and GF mice 1 day prior to delivery, the proportions of CD11b^+^Gr-1^+^ cells were comparable and high (25–30%), confirming that during gestation this myeloid progenitor cell subset is abundant in the spleen 27.

Due to the rapid differentiation and migration of a vast number of hematopoietic cells, early life represents an extremely complex and dynamic period, where many events take place concomitantly. The diversity and dynamics of the CD11b^+^Gr-1^+^ cells present in the spleen during these days reflect this very well and suggest that both influx and differentiation, and perhaps also efflux of cells, take place in the spleen. The CD11b^+^Gr-1^+^ cells most likely comprised different groups of differentiating cells of the myeloid lineage 33. Especially around birth (PND-1 to PND1) we found dramatic changes in distribution. But while the cells of GF mice became smaller in this period, cells of CONV mice sustained CD11b^+^Gr-1^+^ splenocytes of different sizes during the first days of life. Noteworthy, the three groups of CD11^+^Gr-1^+^ cells distinguished by size were already distinct in the fetal CONV spleen, suggesting an influence of the microbiota already during gestation. Regardless of this, the overall proportions of CD11b^+^Gr-1^+^ cells were similar and high (25–30%) in spleens from fetuses taken at the day before expected delivery (PND-1) from CONV and GF mice. This confirms other studies 27 showing that HSCs establish in liver as well as the spleen during gestation. Importantly, this also indicates that an efflux of CD11b^+^Gr-1^+^ cells takes place in the liver at birth leading to a decrease in the CD11b^+^Gr-1^+^ population, which in the colonized mice is counteracted by an influx of similar cells into the spleen, most likely originating from the liver. *Cxcl2* (MIP-2) may hold dual roles; transient up-regulation leads to rapid up-regulation of CD11b on HSC and initiates their differentiation 21 and may furthermore recruit cells from one compartment to another. In the present study, we found a transient up-regulation of *Cxcl2* expression at PND1 in the liver, and both stromal cells, endothelial and the leukocyte cells might be candidate producers of *Cxcl2*. Independently of the source, the up-regulated expression of *Cxcl2* could very well be a key event in both differentiation and recruitment of HSCs from the liver.

Our results thus clearly demonstrate that microbial colonization impacts the perinatal hematopoiesis as well as the early development of adaptive immunity. It is, however, unclear how the colonizing microbes stimulate hematopoiesis, that is, whether direct contact between HSCs and microorganisms takes place and if, where the cells and the microbes encounter. Regardless of the site of encounter, the microbes must trespass the skin or epithelial barrier. The presence of bacteria in the spleen of healthy mice was recently reported by Puga and co-workers 11, hence translocation of bacteria from the GI tract into the blood circulation, and from here to the spleen, is plausible, in particular in the early postnatal period where the GI epithelium is immature. In this regard, we show that intestinal expression of the three genes *Tjp1*, *JamA*, and *Cdh1* exhibited significantly different kinetics in the GF neonate intestine compared to the CONV neonate intestine from PND1 to 6, pointing to a microbiota-dependent acceleration of the maturation. We speculate that the microorganisms present in the gut after birth may control the postnatal period in which uncontrolled microbial translocation is possible, thus limiting bacterial influx to the very first days of life. Of note, GI epithelium of the MC mice exhibited expression kinetics of the tight junction genes, which were only slightly advanced compared to that of the GF mice. Thus, if this indicates a slower maturation and closure, more bacteria but only of one particular strain would translocate into circulation. Nevertheless, the proportion of CD11b^+^ cells and CD4^+^ T cells in the MC spleen at PND4, 7, and 35 was highly similar to the proportions in spleens of GF mice. Hence, we suggest that a single strain of bacteria is not enough to stimulate HSC differentiation into CD11b^+^Gr-1^+^ cells. This is supported by the far stronger MIP-2 response observed from neonate spleen cells stimulated with a mixture of Gram positive and Gram negative bacteria compared to the response upon stimulation with a single strain of bacteria. Notably, at PND35 but not earlier, a significantly lower total number of leukocytes was found in the GF spleens compared to spleens from MC and CONV mice. Thus, the mono-colonization seems to be sufficient to stimulate growth of the spleen to the same rate as seen in conventional colonization, but not to accelerate the number of CD4 cells beyond the GF level.

CD11b^+^Gr-1^+^ cells comprise a heterogeneous group of myeloid-derived cells, which includes differentiating (immature) cells as well as mature neutrophils 16,28,29 and share the lobed nuclei of mature neutrophils 30. In mice, immature CD11b^+^Gr-1^+^ cells are often referred to as myeloid-derived suppressor cells (MDSC) and represent a subset of cells known to expand in blood and lymphoid organs during cancer, inflammatory conditions, and infection holding immunosuppressive properties 17,20. Based on expression of CD11b, Gr-1 and scatter distribution, we described a heterogeneous nature of these cells, comprising at least three distinct subpopulations in fetal and neonatal spleens, which differed between CONV and GF splenocytes, indicating a microbial influence on immune cell development already during gestation. With the well-established importance of a microbiota for proper development of a balanced immune system 3,4 in mind, the observed prolonged presence of a high proportion of CD11b^+^Gr-1^+^, only in spleens of neonate mice born by dams with a diverse microbiota, might suggest a key role for these cells in proper development of the adaptive immunity. To establish this, however, requires assessment of the subpopulations of CD4^+^ lymphocytes around PND7 to PND35, a task which is beyond the scope of the present study, where we aimed to describe the very early microbiota-dependent postnatal cellular events.

In conclusion, we have presented data showing that the microbiota affects homeostatic events of importance for maturation of the splenic CD4^+^ T cell pool, and that CD11b^+^Gr-1^+^ neutrophil-like cells are highly abundant in the neonatal spleens of CONV mice but not in GF or MC spleens. Even though we cannot from these data precisely pinpoint the mechanisms by which the CD11b^+^Gr-1^+^ cells are increased in the spleens of pups from CONV dams, the demonstration of the vast difference in the number of these cells PND2–7 strongly supports the key role of the microbiota from the very first period postpartum. We have thus identified an important perinatal microbiota-dependent event that may impact the subsequent population and polarization of T cells in the neonatal spleen in mice.

## Materials and Methods

### Preparation of bacterial inoculum

*L. acidophilus* NCFM (Danisco, Copenhagen, Denmark) aliquots were prepared by inoculation in de Man, Rogosa, and Sharpe broth (MRS) (Merck, Darmstadt, Germany) for anerobic growth overnight at 37 °C. The culture was harvested by centrifugation, washed in sterile PBS (Lonza, Basel, Schwitzerland), and diluted in PBS to 5 × 10^8^ CFU/ml. Plate counts were performed on MRS agar. The culture was frozen at −80°C until use.

### Animals and tissues

GF and CONV Swiss Webster mice, purchased from Taconic (Lille Skensved, Denmark), were bred and housed in sterile isolators or under specific pathogen-free conditions, respectively. GF mice were treated as previously described 13. Eight female and two male GF mice were mono-colonized with *L. acidophilus* NCFM by applying 5 × 10^8^ CFU/ml in 0.5 ml PBS suspension orally and 0.5 ml to the abdominal skin. To confirm sterility and mono-colonization, respectively, fecal samples from GF and NCFM mice were cultured weekly on non-selective Luria-Bertani medium, under aerobic and anerobic conditions. The day of birth was identified as PND1, and four pups of each group were euthanized at PND1, 2, 4, 7, 14, and 35 and spleens dissected. Furthermore, spleens from two GF and two CONV litters, taken by caesarian section at gestational Day 19 (identified as PND-1) were dissected. Single cell suspensions were prepared of all spleens. Additionally, at PND2, 4, and 7, spleens and livers from GF and CONV pups were formalin-fixed for preparation of tissue sections.

For gene expression analysis, liver and distal ileum from eight CONV, eight MC, and eight GF pups, delivered by four different dams of each group, were dissected and frozen in RNAlater (Qiagen, Hilden, Germany). All animal experiments were approved by the Danish Council for Animal Experimentation.

### Preparation of splenic single cell suspensions

Spleen cells were meshed through a filter, washed and centrifuged in cooled RPMI 1640 with penicillin and streptomycin (Lonza). Five minutes lysing of erythrocytes by suspension in 0.83% ice-cold ammonium–chloride preceded a final washing step and resuspension in RPMI. Viable cells were measured by Nucleocounter® NC-100™ (Chemometec, Allerød, Denmark).

### Immunostaining and flow cytometry

Spleen cell surface antigens were analyzed by use of antibodies: Anti-CD3-PE clone 145-2C11, anti-CD4-APC clone RM 4-5, anti-CD8-APC clone 53-6.7, anti-CD11b-PECy7 clone M1/70, anti-Ly6G(Gr-1)-APC clone RB6-8C5, and anti-F4/80-FITC clone BM8 (all eBioscience, San Diego, CA) after blocking of FC antibody-binding by anti-CD16/CD32 (BD Biosciences, Franklin Lakes, NJ) for 10 min. Cells were fixed in PBS with 2% methanol-free formaldehyde and analyzed within 1–3 days on a FACScanto flow cytometer (BD Biosciences). Data analyses and layouts were performed using FlowJo V10 (Tree Star, Ashland, OR).

### RNA-isolation and real-time quantitative PCR (qPCR)

Ilea and livers kept in RNAlater were removed from storing solution and homogenized in RLT buffer from Qiagen (Hilden, Germany). RNA extraction, quantification, quality evaluation, reverse transcription, primer testing and validation, and data analysis were carried out as previously described 31. *Cxcl1*, *Cxcl2*, and *Arg1* were analyzed with TaqMan® gene expression assays; Mn00433859_m1(*Cxcl1)*, Mn00436450_m1(*Cxcl2)*, Mn00475988_m1(*Arg1*), and Mn006007939_s1(*Actb*) (Life Technologies, Carlsbad, CA). The following primers were used for analyses of tight junction genes; *Cdh1*-forward GTATCGGATTTGGAGGGACA, *Cdh1*-reverse CAGGACCAGGAGAAGAGTGC, *JamA-*forward GTTCCCATTGGAGTTGCTGT, *JamA*-reverse GGGAGAGGAGAAGCCAGAGT, *Tjp1*-forward GGTGACATTCAAGAAGGGGA (designed with NCBI PrimerBlast), *Tjp1*-reverse TCTCTTTCCGAGGCATTAGCA 32, *Actb*-forward GTCCACCTTCCAGCAGATGT, and *Actb*-reverse GAAAGGGTGTAAAACGCAGC 31.

### Ex vivo stimulation of spleen cells

Production of MIP-2 was measured upon addition of *E. coli* Nissle 1917 (MOI 4), *L. acidophilus* NCFM (MOI 1.5), or half the amount of each bacteria in a mixture to 5 × 10^5^ spleen cells in 0.1 ml RPMI. Supernatants were harvested after 20 h (37 °C) and concentrations estimated using a commercial MIP-2 ELISA kit (R&D systems, Minneapolis, MN).

### H&E staining

Spleens and livers were fixed in 4% buffered formalin, embedded in paraffin, and sliced into 5 μm sections. The tissues were mounted on SuperFrost Plus slides (Menzel-glaser, Braunschweig, Germany) and stained by H&E for evaluation of cellular composition. For quantification of granulocytic cells with polyloped or ringed nuclei in each animal, five random fields of view at 63× magnification for each sample were counted blinded and independently by two trained persons. Samples were analyzed by Student's *t*-test (unpaired, two-tailed) for statistically differences between age and/or treatment.

### Statistics

Statistics were performed with GraphPad Prism™V5.03 (GraphPad Software, Inc., La Jolla, CA).

## Supporting Information

Figure S1 : Perinatal CD11b^+^Gr-1^+^ spleen cells comprise three distinct subpopulations differing in size and brightness of markers CD11b and Gr-1.
