# Supplementary figures and images for: Neonatal microbial colonization in mice promotes prolonged dominance of CD11b+Gr-1+ cells and accelerated establishment of the CD4+ T cell population in the spleen
Source: Immun Inflamm Dis. 2015 Jun 18;3(3):309–20. doi: 10.1002/iid3.70 (PMC4578529; doi:10.1002/iid3.70)

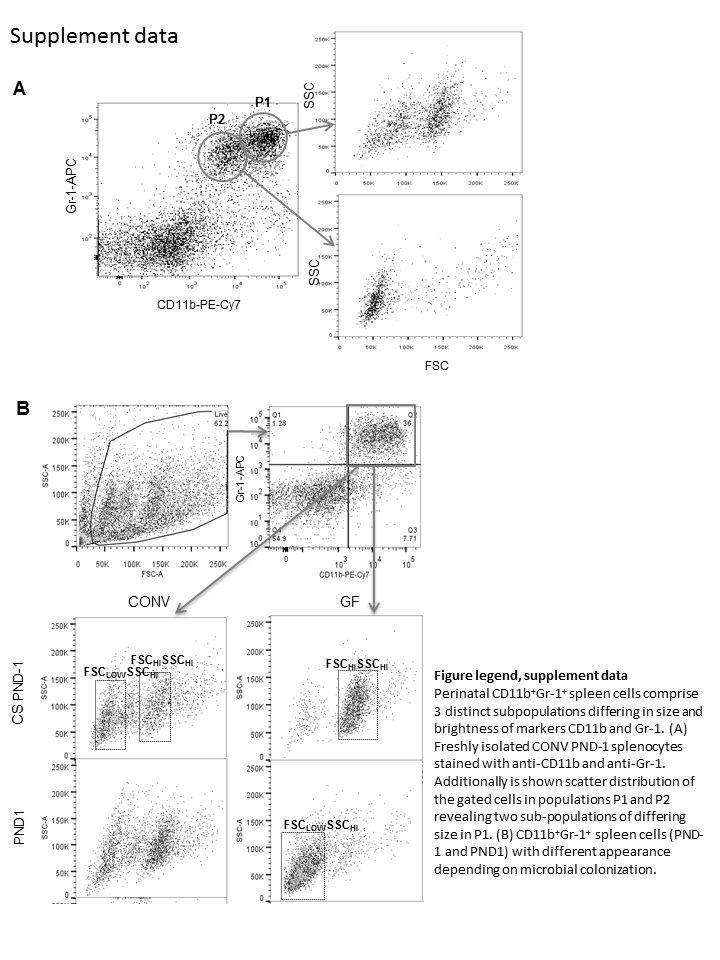

Supplement: Figure S1 — : Perinatal CD11b+Gr-1+ spleen cells comprise three distinct subpopulations differing in size and brightness of markers CD11b and Gr-1. [file iid30003-0309-sd1.tif]
